# Sickness absence transitions among Swedish twins with back, neck or shoulder pain and common mental disorders applying a multi-state approach

**DOI:** 10.1038/s41598-023-37572-5

**Published:** 2023-06-29

**Authors:** Sanna Kärkkäinen, Jakob Bergström, Annina Ropponen, Mo Wang, Jurgita Narusyte, Pia Svedberg

**Affiliations:** 1 Division of Insurance Medicine, Department of Clinical Neuroscience, Karolinska Institutet, Stockholm, Sweden; 2 Finnish Institute for Health and Welfare, Helsinki, Finland; 3 Finnish Institute of Occupational Health, Helsinki, Finland

**Keywords:** Psychology, Environmental social sciences, Diseases, Medical research, Risk factors

## Abstract

We aimed to investigate transitions to and from sickness absence, or disability pension among individuals with back, neck, or shoulder pain and/or with common mental disorders (CMDs), and the role of familial (genetics and shared environment) influences on the transitions. Swedish twins born 1935–1985 who responded to pain and CMDs survey items (N = 41,516) were followed on average 8.7 years for sickness absence states in national registers. Multi-state Cox regression models were applied for three exposure groups: pain, CMDs, and presence of both, compared to unexposed. Exposure discordant twin pairs, stratified by zygosity, were analysed to assess the role of familial factors. Hazard Ratios (HR) with 95% confidence intervals and transition intensities were calculated. HRs were similar for transitions between states among those with pain or CMDs. The highest HRs were for transitions from entry to sickness absence and sickness absence to disability pension among those with both pain and CMDs (HRs: 1.61 and 1.43, respectively). Higher HRs for dizygotic compared to monozygotic twins for the first transition to sickness absence and for altering back to not being sickness absent indicate familial confounding. Back, neck, or shoulder pain and/or CMDs indicate a higher risk to become sickness absent and for repeated sickness absence episodes over time compared to unaffected.

## Introduction

Pain at various sites of the body and common mental disorders (CMDs) i.e., depression and anxiety are common in the working age population^1,2^. These symptoms may contribute to, besides individual suffering, sickness absence (SA) or even disability pension (DP)^2^. But knowledge is still scarce regarding the influential factors on transitions between SA, not being sickness absent and considered being available for the labor market, or to DP among those with pain or CMDs. Beside age, sex, health-, and socioeconomic status, also familial factors (genetics and shared childhood family environment) may influence the transitions to and from SA since previous research has shown genetics to be important for musculoskeletal pain^3–5^, CMDs^6^ as well as for SA and DP^7–10^. Hence, a longitudinal twin study assessing transitions (shifts) to and from SA*,* or to DP would be merited.

The overall prevalence of chronic pain at various sites among adults is estimated to be 31%^11^ and pain at one or multiple sites predicts future DP, especially due to musculoskeletal disorders^12–14^. Musculoskeletal pain at one site has also shown to be commonly accompanied by pain in other sites; shoulder pain was more likely to be accompanied by pain in neck and hand area, while a main complaint in the neck area was accompanied by pain in low and upper back^15^. Moreover, SA due to depression has shown to increase the risk of future DP due to any diagnosis, but to a higher degree for DP due to CMDs^12^. Pain in co-occurrence with CMDs vary, yet the evidence seems univocal that co-occurrence is relatively common^16–18^. However, studies of co-occurrence in relation to SA and DP have shown mixed results^19–21^.

Even though some knowledge gaps still exist genetics seem to play an important role for musculoskeletal pain^4,5^ and for DP due to musculoskeletal disorders^8^. For CMDs genetic influences are more well-known showing a moderate influence for depression and anxiety^6^, as well as for SA^22,23^, DP in general^7,8,10^ and for DP due to mental disorders^8,9^. Previous studies also suggest genetic factors to play a role in the association between musculoskeletal pain and CMDs^17,24,25^ as well as for SA due to back pain and CMD with differences by sex when the outcome event was DP^24^.

Transitions between SA, work, DP, unemployment, and mortality, using multi-state models has previously been applied^26–29^. This extension of the traditional survival analysis has shown to be a flexible framework for investigating work life transitions over time. Some studies also investigated the influence of various covariates in this framework, such as age, income, health status, and type of diagnosis^26,29–35^. None of these studies were based on a twin sample to investigate the role of familial factors on the transitions between SA states. More knowledge is also needed on how the transitions to and from SA differ between individuals with musculoskeletal pain and/or CMDs since this is still poorly understood.

The aim was to investigate if there are differences in SA transition probabilities between individuals with back, neck, or shoulder pain and/or with CMDs, and to examine the influence of familial factors (genetics and shared environment) on the transitions.

## Methods

### Source population and selection criteria

This study is based on twins from the Swedish Twin project Of Disability pension and Sickness absence (STODS)^36,37^. Twins were identified through the Swedish Twin Registry (STR) and those who participated in the Screening Across the Life-Span Twin (SALT)-telephone interview conducted between years 1998 and 2003^38^, or the Study of Twin Adults: Genes and Environment (STAGE) conducted between years 2004 and 2006 by STR, were included^39^. Zygosity was determined with questions on similarity of twin pairs in childhood and this method has been shown accuracy of 98% or higher with genetic test in two sub-samples of the STR^38^. From the source population, individuals below the age of 65, alive, living in Sweden and not granted DP or SA when responding to surveys (baseline), or the year before, were included. Age at baseline was calculated from date of birth and survey response date and used for exclusions.

### Register data

Register data were from three national sources: SA spells (> 14 days) and DP from 1994 until 31st December 2013 from the MicroData for Analyses of Social insurance (MiDAS) database at the Swedish Social Insurance Agency (SIA). All individuals in Sweden with an income from work or social benefits above the age of 16, are covered by the national sickness insurance scheme for SA, and all above age 19 for DP. SA and DP benefits covers up to 80% and 65% income lost respectively in case of work incapacity due to medical reasons^37,40^. Shorter SA spells (< 15 days) are not compensated by SIA, hence not included in this study. From Statistics Sweden the Longitudinal Integration Database for Health Insurance and Labour Market Studies Register (LISA) information on socio-demographic variables, including education and emigration, were included^41^. Date of death was available from the National Board of Health and Welfare, Causes of Death register.

### Study population

**Figure 1:**
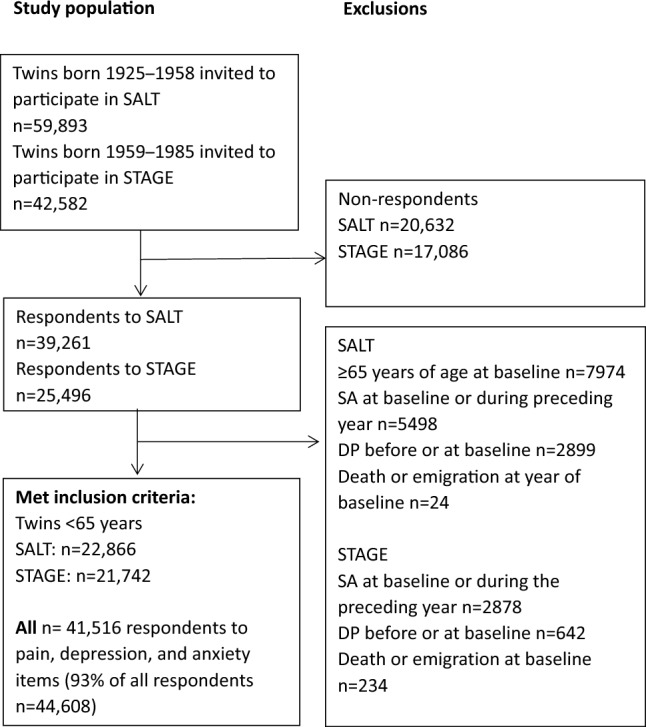
Study population, inclusion, and exclusion criteria. *SALT* screening across the life-span twin study, *STAGE* the study of twin adults: genes and environment, *SA* sickness absence, *DP* disability pension.

The sample included 13,886 complete twin pairs, of these 4682 were monozygotic (MZ), 4434 same-sex dizygotic (DZ), and 4477 opposite-sex DZ, and 293 pairs of unknown zygosity. Additionally, there were 16,836 single individuals without information from the twin sibling due to non-participation. This study included 41,516 individuals [93% of all 44,608 (51% women)] responders to either SALT or STAGE survey items regarding pain sites, depression, and anxiety. Inclusion and exclusion criteria are presented in Fig. 1.

### Exposures

Pain sites and CMDs (depression and anxiety) were assessed at baseline in the surveys (SALT and STAGE).

*Pain* was measured with the question “Do you have or have you had back-, neck-, or shoulder joint pain” (coded as yes/no to each item). Pain was considered as exposure if at least two different pain sites were reported [yes/no for having back, shoulder or neck pain (≥ 2 pain sites)].

*CMD*s were measured based on whether participant had experienced major depression (MD) and/or generalized anxiety symptoms (GAD) (yes/no). Measures were similar in SALT- and STAGE- surveys and DSM-IV was the basis for definitions. In the SALT-questionnaire, criteria for MD and GAD were defined with Composite International Diagnostic Interview-Short Form (CIDI-SF) adapted from DSM-IV. The definition for MD included (a) having dysphoria or anhedonia and depression score of 4 or higher, and/or (b) respondent having used antidepressants. For GAD, at least 1 month of excessive worry and anxiety was required and having at least one out of five DSM-IV symptoms: restlessness or feeling keyed up or on edge, being easily fatigued, irritability, muscle tension, sleep disturbances (difficulty falling or staying asleep, or restless unsatisfying sleep). For a more detailed description see Kendler et al.^42^. In the STAGE survey, the definition for MD was based on Structured Clinical Interview for DSM-IV disorders (SCID). The definition for MD included having dysphoria or anhedonia and depression score of 4 or higher. For GAD, having at least three out of six DSM-IV symptoms was required: feeling restlessness or feeling keyed up or on edge, being easily fatigued, having difficulty concentrating or mind going blank, irritability, muscle tension, sleep disturbances queried as difficulty falling or staying asleep. For detailed description, see Mather et al.^43^.

The exposure variable was coded as (1) *Unexposed* (≤ 1 pain site/no CMDs); (2) *Pain* (≥ 2 pain sites); (3) *CMDs* (depression and/or anxiety); (4) *Both* pain and CMDs.

In addition, a binary variable exposed/unexposed to pain and/or CMDs was created.

### Covariates

*Sex* was a dichotomous variable (women/men). *Birthyear cohorts* were created and used, categorized into 10-year periods based on birthdate: 1935–1944, 1945–1954; 1955–1964, 1965–1974, 1975–1985. There was no overlap of individuals between SALT and STAGE-participants. *Education* was determined from LISA information coded as primary education (9 years), secondary education (12 years) and higher education (> 12 years). From survey data *marital status* was dichotomized to those not being married or cohabiting, and those married/cohabiting, and *self-rated health* was categorised into good (excellent or good), moderate, and poor (not so good or poor) health.

### Sickness absence states

**Figure 2:**
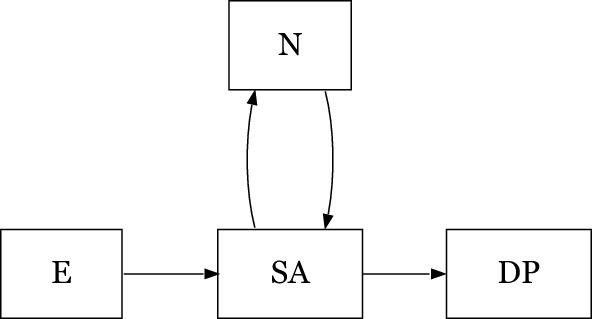
States and possible transition. *E* Entry/baseline i.e., date of survey response (not on SA or DP), *SA* sickness absence, *N* not sickness absent, *DP* disability pension.

States in this study were: entry [E] (baseline, not on SA or DP at entry or during the preceding year); received SA benefits, either full or partial, due to any diagnosis [SA]; not on SA (considered available for the labor market) [N]; and being granted DP (absorbing state) [D] (Fig. 2). During the follow-up, an individual may shift between states of being on SA and N (not on SA).

### Statistical analyses

Descriptive statistics were used, and multi-state models were applied to investigate individuals’ transition between states and the events of interest were the transition probabilities between the states included. Since this study included data from two surveys with participants of wide age span, adjustment for potential cohort effects was done by using birth year categories. All the analyses were also adjusted for sex.

In the multistate-model, transitions between states were expressed by hazard functions for all possible transitions (see^28,44^). A semi-parametric reversible multi-state Cox’s regression model was used to evaluate the effect of exposure on the following transitions:

- Study baseline/entry, transition to sickness absence (E → SA).
- Not sickness absent (available for the labor market), transition to sickness absence (N → SA).
- Sickness absence, transition to Not being sickness absent (available for the labor market) (SA → N).
- Sickness absence, transition to disability pension (SA → DP).

All individuals were considered employed, unemployed or otherwise out of the labor market but i.e., available to the labor market at study onset meaning that the transition E to SA is the hazard for a first SA spell given that an individual has been free of SA and DP at baseline and 1 year prior to study baseline. N to SA is therefore the hazard of transitions to SA from being employed/available for the labor market given that an individual already has had one SA after study onset. Individuals were allowed to enter N and SA multiple times, but when entering DP, no going back from that state was allowed. Thus, DP was an absorbing state. Due to low numbers of individuals who died or emigrated these events were also absorbing states but due to model convergence problem we decided not to include those states in our multi-state model. During follow-up censoring was applied for old-age pension and age > 65 years. Transition probabilities with 95% confidence intervals (CIs) and state occupation probabilities were estimated using the Aalen–Johansen estimator^45^.

#### Transition probabilities by sickness absence states and exposure groups

All individuals were included in the models to investigate transition probabilities between the states by exposure groups: pain, CMDs or both, using unexposed as reference. Two multi-state analyses were performed including all observations (using twins as singletons). The first model included exposure, sex and birth cohort categorized into 10-year as covariates. The second model included additionally education, self-rated health, and marital status. The units for person time were counted as days. Using time as clock-forward (real time) require that transitions to a state is only affected by the present state and future ones. The Markov chain assumption was tested by adding total time spent in state N as a covariate in a model with the transition SA to DP as all individuals are assumed to go from N to SA before experience DP. Total time spent in N had a clear effect on the transition SA to DP therefore time was incorporated into the analysis as clock-reset (time set to zero after any transition occurred). The multi-state model was stratified on transition, and transition covariate specific analyses was performed investigating the effect of exposure on each transition.

#### The influence of familial factors on sickness absence transitions

Applying the multi-state models to twin data allows to investigate if the transition probabilities are confounded by familial (genetics and shared environment) factors. A difference in transition probabilities between states for MZ and DZ same sex twins would indicate the importance of familial factors. Hence, to investigate the influence of familial factors on the transitions between states the exposure was dichotomized (due to lower number of complete pairs available) into unexposed (no pain/only one pain site or no CMDs at baseline) and having any of the disorders/symptoms (≥ 2 pain sites or CMDs, or both). Number of exposure discordant twin pairs were: 1298 MZ and 1555 DZ of same sex. MZ and DZ twin pairs were analyzed in separate models and combined. Robust standard errors were used when estimating 95% CIs due to the dependency between twins in pairs. We also performed a conditional multi-state Cox regression model stratifying on twin pairs and transition.

#### Transition probabilities with dichotomized exposure

Transition probabilities were calculated for the whole cohort using the dichotomized exposure variable.

The analyses were performed using the mstate package^46^ in the statistical software R (version 4.1.2). https://CRAN.R-project.org/package=mstate (Accessed 9th Feb 2022)^47^.

### Ethical considerations

The study was approved by the Regional Ethical Review Board in Stockholm (2007/524-31; 2010/1346-32-5; 2014/311-32; 2017/128-32) and follow the Helsinki Declaration of 1975, as revised in 1983. Informed consent was obtained from all participants in the STAGE and SALT surveys.

## Results

Among those who participated in the SALT or STAGE survey 93% (41,516/44,608) responded to both pain and CMD items (99% in SALT 22,744/22,866 and 86% in STAGE 18,769/21,742). Mean age at baseline was 43.5 years (SD 12.1). Descriptive statistics at baseline for all individuals by exposure group; pain (≥ 2 pain sites), CMD, pain and CMD, and unexposed (no CMDs, ≤ 1 pain site) are shown in Table 1. Those who had no pain or CMDs reported better self-rated health compared to those who reported pain or CMDs. University education was more common among those unexposed or with only CMDs as compared to those who reported pain or had combined pain and CMDs. Older birth cohorts reported more often pain and CMDs compared to younger cohorts.

**Table 1:** Descriptive statistics for the whole sample (n = 44,608) and for the responders to survey items regarding at least two pain sites (back, neck or shoulder) and CMDs (depression, anxiety) (n = 41,516) by exposure group.

	Total (N = 44,608)	Unexposed (no CMD or Pain) (n = 28,261)	CMD (n = 4,964)	Pain (n = 6,322)	Pain and CMD (n = 1,969)
Sex					
Men	21,864 (49%)	14,812 (52%)	1767 (36%)	3034 (48%)	627 (32%)
Women	22,744 (51%)	13,449 (48%)	3197 (64%)	3288 (52%)	1342 (68%)
Survey					
SALT	22,866 (51%)	14,142 (50%)	3310 (67%)	3903 (62%)	1392 (71%)
STAGE	21,742 (49%)	14,119 (50%)	1654 (33%)	2419 (38%)	577 (29%)
Birthyear cohorts					
1935–1944	8183 (18%)	5217 (18%)	987 (20%)	1476 (23%)	442 (22%)
1945–1954	10,608 (24%)	6417 (23%)	1664 (34%)	1793 (28%)	693 (35%)
1955–1964	9016 (20%)	5524 (20%)	982 (20%)	1454 (23%)	438 (22%)
1965–1974	8169 (18%)	5261 (19%)	531 (11%)	1053 (17%)	227 (12%)
1975–1985	8632 (19%)	5842 (21%)	800 (16%)	546 (9%)	169 (9%)
Education					
University	16,923 (38%)	11,180 (40%)	2177 (44%)	1699 (27%)	605 (31%)
High school	20,762 (47%)	12,880 (46%)	2095 (42%)	3220 (51%)	997 (51%)
Elementary school	6904 (15%)	4187 (15%)	691 (14%)	1400 (22%)	367 (19%)
Missing	19 (0%)	14 (0%)	< 5 (0%)	< 5 (0%)	0 (0%)
Self-rated health					
Good	36,836 (84%)	25,162 (89%)	3901 (79%)	4720 (75%)	1245 (63%)
Moderate	5964 (14%)	2800 (10%)	867 (17%)	1381 (22%)	554 (28%)
Poor	915 (2%)	289 (1%)	192 (4%)	217 (3%)	165 (8%)
Missing	893 (2%)	10 (0%)	< 5 (0%)	< 5 (0%)	5 (0%)
Martial status					
Married/cohabit	31,762 (72%)	20,629 (73%)	3152 (64%)	4925 (78%)	1339 (68%)
Not married/cohabit	12,252 (28%)	7482 (27%)	1788 (36%)	1378 (22%)	625 (32%)
Missing	594 (1%)	150 (1%)	24 (1%)	19 (0%)	5 (0%)
Zygosity					
MZ	13,573 (30%)	8798 (31%)	1466 (30%)	1837 (29%)	551 (28%)
DZ	14,728 (33%)	9450 (33%)	1664 (34%)	2121 (34%)	662 (34%)
Unknown	1119 (3%)	575 (2%)	95 (2%)	121 (2%)	35 (2%)
DZOS	15,188 (34%)	9438 (33%)	1739 (35%)	2243 (35%)	721 (37%)

*MZ* monozygotic, *DZ* dizygotic, *DZOS* opposite sex dizygotic, *SALT* screening across the life-span twin study, *STAGE* the study of twin adults: genes and environment, *CMD* common mental disorders (depression, anxiety).

### Transition probabilities by sickness absence states and exposure groups

In total, 39% percent of the total study population transferred to SA at least once during follow-up (Table 2). There were few transitions directly to DP from entry and from N (not on SA/returned from SA) to DP. Follow-up time was on average 8.7 years [standard deviation (SD) 2.7] (0–13.3 years).

**Table 2:** Number of transitions (shifts) between states among participants (n = 41,516) during follow-up by exposure group (Unexposed, CMDs, Pain (at least two pain sites), pain and CMDs).

Transitions (from-to)	Unexposed (n = 28,261)	CMD (n = 4964)	Pain (n = 6322)	Pain and CMD (n = 1969)
E (no transition to SA or DP)	18,370	3126	2501	754
E → DP	69	23	33	16
E → SA	9822	3173	2430	1199
N → DP	58	29	28	11
N → SA	7618	3360	2808	1554
SA → DP	727	417	266	195
SA → N	16,151	5923	4788	2479
Total n of transitions	52,815	16,051	12,854	6208

States and possible transition: *E* entry/baseline i.e., date of survey response (not on SA or DP), *SA* sickness absence, *N* not sickness absent, and *DP* disability pension.

**Figure 3:**
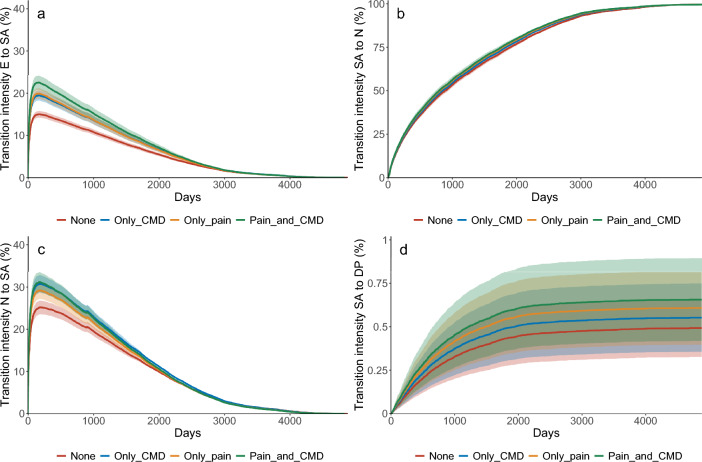
Transition intensities (probabilities) as a function of time in days for unexposed and exposed to pain, CMDs (depression, anxiety), or both. *E* entry, *SA* sickness absence, *N* not sickness absent, *DP* disability pension. *Note*: The scale on the *y*-axis differs depending on transition investigated.

Results from the multi-state models including all individuals by exposure group (pain, CMDs, both pain and CMDs) with adjustment for sex and birth year cohort (model 1) and from the fully adjusted model (model 2) are presented in Table 3. In the full model, HRs were similar for transitions E to SA, N to SA and SA to N for those with pain and CMDs, respectively. The risk of transitioning from SA to DP was higher among those reporting pain (HR; 1.30 [95% CI 1.15–1.47]) than those reporting CMDs (HR; 1.16 [CI 1.00–1.34]). The highest HRs were found for transitions E to SA and SA to DP among those with both pain and CMDs (HRs: 1.61 and 1.43, respectively). The transition intensities (probabilities) were higher among those with both pain and CMDs for the transition to a first SA, from SA to DP, and somewhat higher for moving from N to SA, while the intensities were similar transitioning from SA to N (Fig. 3).

**Table 3:** Hazard ratios (HR) with 95% confidence intervals (CI) for each transition for the whole study population (N = 41,516) by exposure group: pain (at least two pain sites), CMD, and both and no pain or CMD (unexposed) as reference.

Exposure	Transition	Model 1		Model 2	
		HR	95% CI	HR	95% CI
Pain					
	E → SA	1.49	1.43–1.55	1.39	1.34–1.45
	N → SA	1.27	1.21–1.34	1.22	1.16–1.28
	SA → N	1.11	1.07–1.15	1.09	1.05–1.13
	SA → DP	1.50	1.33–1.69	1.30	1.15–1.47
CMD					
	E → SA	1.35	1.29–1.41	1.35	1.29–1.41
	N → SA	1.31	1.24–1.38	1.29	1.22–1.36
	SA → N	1.08	1.03–1.12	1.07	1.02–1.11
	SA → DP	1.24	1.07–1.42	1.16	1.00–1.34
Pain and CMD					
	E → SA	1.75	1.65–1.85	1.61	1.52–1.71
	N → SA	1.42	1.33–1.51	1.33	1.25–1.42
	SA → N	1.16	1.10–1.23	1.12	1.06–1.19
	SA → DP	1.77	1.50–2.08	1.43	1.21–1.69

Follow-up time was from baseline (entry) until 31 December 2013.

Model 1: adjusted for sex and birthyear cohort. Model 2: adjusted for sex, birthyear cohort, education, self-rated health, and marital status. *E* entry, *SA* sickness absence, *N* not sickness absent, *DP* disability pension. The reference group are those unexposed to pain and CMDs (depression, anxiety).

Reviewing the multi-state model results with all covariates revealed that women, earlier birth cohort (born before year 1954), poor self-rated health, and low education were risk factors for transitioning from baseline to SA and from N to SA (HRs: 1.31–1.48). The highest risk for transitioning from SA to DP was observed for the oldest birth year cohort (born 1935–1944) (HR: 25.00 [95% CI 16.50–38.00]). No or only small effects of marital status on the transitions was shown (data not shown in table).

### The influence of familial factors on sickness absence transitions

**Figure 4:**
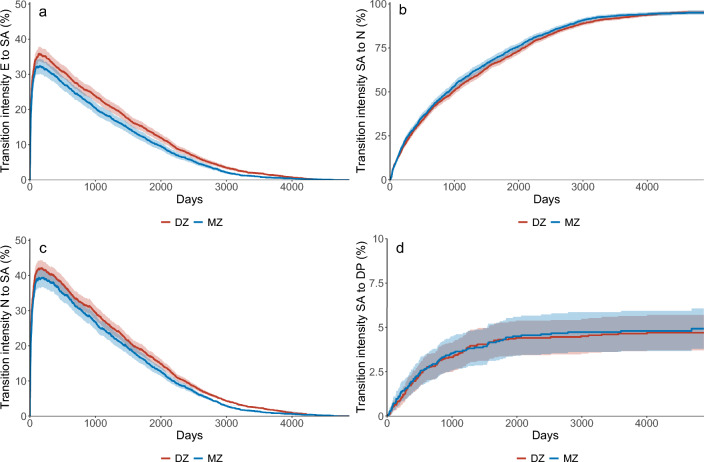
Transition intensities (probabilities) as a function of time in days for monozygotic (MZ) and same-sex dizygotic (DZ) twin pairs using binary exposure variable (Pain, CMDs, or both/unexposed). *E* entry, *SA* sickness absence, *N* not sickness absent, *DP* disability pension. *Note*: The scale on the *y*-axis differs depending on transition investigated.

The results of the conditional multi-state analysis of complete same-sex twin pairs showed larger HR between exposed and non-exposed for DZ compared to MZ when twins experienced the first transition to the SA state from entry (HR DZ: 1.43 [95% CI 1.29–1.58], HR MZ: 1.16 [95% CI 1.04–1.29]) and same pattern was shown for the reversible states N to SA. HRs were similar and non-statistically significant for DZ and MZ twin pairs for the transitions SA to N and for the transition to the absorbing state DP (Table 4). Figure 4 plots the transition intensities, extracted from the multi-state analysis, as a function of time and show that the probabilities are higher for DZ compared to MZ regarding the transition to a first SA and for the probability moving from N to SA. Results (Table 4, Fig. 4) suggest that genetic factors influence experiencing a first SA after baseline, less when the reversible process begins (≥ 2 SA episodes), and not at all when SA leads to DP.

**Table 4:** Hazard ratios (HR) with 95% confidence intervals (CI) for each transition for the exposure discordant complete same-sex twin pairs with binary exposure: pain (at least two sites) and/or CMD and unexposed as reference.

Exposure	Transition	DZ (n = 1555 pairs)		MZ (n = 1298 pairs)		All (MZ + DZ) (n = 2853 pairs)	
		HR	95% CI	HR	(95% CI)	HR	(95% CI)
Pain and/or CMD	E → SA	**1.43**	**1.29–1.58**	**1.16**	**1.04–1.29**	**1.30**	**1.19–1.43**
	N → SA	**1.19**	**1.04–1.35**	1.13	0.99–1.28	**1.22**	**1.08–1.38**
	SA → N	1.07	0.97–1.17	1.07	0.97–1.18	1.08	0.98–1.20
	SA → DP	1.00	0.70–1.42	1.09	0.76–1.56	1.09	0.71–1.69

*DZ* dizygotic same-sex twin pairs, *MZ* monozygotic twin pairs. Bold = statistically significant HRs. *E* entry/baseline i.e., date of survey response (not on SA or DP), *SA* sickness absence, *N* not sickness absent, and *DP* disability pension, *CMD* common mental disorders (depression, anxiety).

### Transition probabilities with dichotomized exposure

In the multi-state model including all individuals with the dichotomous exposure variable, the largest HR was observed for the first transition to SA (HR: 1.41 [95% CI 1.36–1.45]) and the reversible transitions showed a higher HR for N to SA (HR: 1.26 [95% CI 1.22–1.32]) than the transition SA to N (HR: 1.09 [95% CI 1.06–1.12]) in the fully adjusted model (Supplementary Table S1). The HR of ending up with DP after SA was also higher for the exposed (HR: 1.28 [95% CI 1.15–1.41]). The results from model 1 adjusting for sex and birth year cohort showed higher HRs compared to the fully adjusted model, especially pronounced for the transition from SA to DP. Reviewing the multi-state model results with all covariates reveals that good self-rated health and higher education lower the risk of transitioning from SA to DP among those with pain and/or CMDs. No effect of marital status on the transition to DP was shown. Hence, a similar risk pattern was seen as for each exposure group separately (Table 2).

In addition, sensitivity analyses applying a frailty model with the binary exposure variable were conducted. Results were almost identical to main results of the conditional model (data not shown). We also stratified the main analysis including all individuals based on survey participation (STAGE or SALT) since data collection time point and birth cohorts differ. These results were also in line with the main results (data not shown).

## Discussion

In this study we investigated probabilities of shifting to and from SA, and to DP, among Swedish twins with a self-reported history of neck, back or shoulder pain, or CMDs (depression and anxiety), or with a combination of pain and CMDs. We found that those with at least two pain sites, or CMDs, or with a combination of pain and CMD, had higher risk of transitioning between different SA states in comparison to those unexposed to pain and/or CMDs. The highest risk estimates were however found for those with both pain and CMDs, especially for the transition from SA to DP, in support of earlier findings^20,21^. Familial (genetics and shared family environment) and other factors seemed to influence the transition hazards to some degree.

Pain but also CMDs were more prevalent in older age groups in the present study and such health problems are known to be common health challenges which may cause obstacles and functional limitations both at work and in leisure time^1,2,48,49^. Previously, there has been few studies and hence sparse knowledge if those with coexisting pain and CMDs have similar or differing risk of transitioning to SA or DP compared to those without such symptoms or compared to those with only pain or CMDs. It was therefore important to investigate if pain and CMDs have informative value regarding risk of SA and DP over time. Given to recent governmental suggestions in the Nordic and other western countries, work participation should be extended in higher ages during the coming decade. By any means to achieve such a goal, transitions from working life to temporary or permanent work incapacity should be identified and any influential factors investigated, also considering various symptoms and disorders.

In this study the risk of transitioning to and from SA states (not on SA, SA, or DP) were relatively similar in the presence of pain sites or CMDs. Hence, pain and mental health symptoms are important for the SA transitions and should be acknowledged in health care and work settings. Our results correspond with other studies showing that individuals with chronic disorders have an increased risk of SA, but also that previous SA increased the risk of transitioning from work to SA, from SA to unemployment, from work to unemployment, and from work to DP^29,33,50,51^. A more recent study also reported that the expected time in SA was longer among employees with depressive symptoms compared to those without such symptoms, while the expected time in work was shorter^35^. This could also be useful information when planning interventions to prevent future SA and DP. It was for example shown in an earlier study that a simple medical intervention among individuals with low back pain did not decrease the probability of being on SA, but the intervention decreased the time individuals were on SA^27^ which is also important for individuals as well as for employers. Hence, early identification and treatment and/or work adjustments may help individuals getting back to work, or even prevent SA in the first place.

Previously identified risk factors for work life expectancy, SA and DP were confirmed also in the present study: women, older age groups, poor self-rated health, or having low education were influential risk factors on transitions from baseline (or recovered from SA episode) to SA^29,33,35^. In addition, using data from a Swedish population-based twin setting, a unique possibility to investigate the importance of familial influences on the development of SA and DP in the presence of pain and/or CMDs was provided in the present study. Results show that familial factors are likely to play a role in shifting between states; being on SA or not, and to the risk of transition to DP. This finding is in line with our hypothesis based on previous research that has shown genetic factors to influence musculoskeletal disorders and pain^3–5^, depression and anxiety^6^, SA^22,23^, and DP^7–10^, and on the association between pain and CMDs^17,25^. Hence, genetic influences on morbidity seem to be reflected in the transition from pain and CMDs to SA and DP, but the environment is also important which opens opportunities for adjustments and interventions targeting work incapacity.

### Strengths and limitations

Strength includes the national register data on SA and DP with no loss to follow-up. Further, the sample size was large including those without SA or DP at baseline or the year before follow-up started. This means, that those with pre-existing severe diseases, or in the process of DP, were excluded. Since this study included twin pairs, it was possible to consider the role of familial influences in addition to sociodemographic factors. Moreover, applying multi-state models to SA data based on a twin sample is to the best of our knowledge a new approach. We investigated familial influences by combining those with back, neck or shoulder pain (at least two pain sites) and/or CMDs (compared to those without), to increase power in the analysis and since results of these symptom groups did not differ much from each other in the main analysis regarding the risk of shifting to and from SA. Limitations include that our findings are based on self-reported upper body musculoskeletal pain sites and CMDs and cannot be used to derive information on medically confirmed and diagnosed disorders. Further, we were unable to incorporate information on pain duration and severity since such information was lacking. These aspects should be addressed in future research as they may influence the prognosis of the disorders including the need of (and duration or frequency) SA and DP. If under- or over-reporting bias in pain sites or CMDs exists, our results still show the association which could be tested in future studies. We defined pain as being present in at least two sites, but pain definitions vary between studies making comparison to other results challenging. Moreover, besides SA and DP, shifting to other benefits were not considered for two main reasons: we do not have access to data of start and end dates for unemployment or other social benefits, and individuals who have social benefits are considered available for the labour market and qualified for SA and DP. Further, no data on work exposures were available and such could also possibly influence the results. Yet another weakness may be that this study was conducted within the Nordic social security model which may affect generalizability to other countries.

## Conclusions

To conclude, individuals who experience musculoskeletal pain and/or CMDs have a higher risk to transition to SA, experience repeated SA episodes, and for shifting to DP, in comparison to those unaffected by pain and CMDs. Our findings suggest that health and demographic factors play a role for the SA transition probabilities in a working age population and familial factors mainly influence the transition to first and recurrent SA.

## Supplementary Information

Supplementary Information.

## Data Availability

The data that support the findings of this study are available from the original sources: the Swedish Twin Registry, Statistics Sweden, the Swedish Social Insurance Agency and the Swedish National Board of Health and Welfare. Restrictions apply to the availability of the data used in this study based on the Swedish Twin project of Disability pension and Sickness absence (STODS), which were used with ethical permission for the current study and therefore are not publicly available. Data are however available from the corresponding authors upon reasonable request and with permission of the Swedish Twin Registry, Statistics Sweden, the Swedish Social Insurance Agency and the Swedish National Board of Health and Welfare.
