# Supplementary material for: Sickness absence transitions among Swedish twins with back, neck or shoulder pain and common mental disorders applying a multi-state approach
Source: Sci Rep. 2023 Jun 29;13:10520. doi: 10.1038/s41598-023-37572-5 (PMC10310811; doi:10.1038/s41598-023-37572-5)
Supplement: Supplementary file 1 — Supplementary Information. [file 41598_2023_37572_MOESM1_ESM.docx]

**Supplement**

**Table S1.** Hazard Ratios (HR) with 95% Confidence Intervals (CI) for each transition for the whole study population with binary exposure: pain and/or common mental disorder (CMD) and no pain or CMD (unexposed) as reference.

| **Exposure** | **Transition** | **Model 1** | | **Model 2** | |
| --- | --- | --- | --- | --- | --- |
|  |  | **HR** | **95 % CI** | **HR** | **95 % CI** |
| Pain and/or CMD | E → SA | 1.47 | 1.42-1.52 | 1.41 | 1.36-1.45 |
|  | N → SA | 1.31 | 1.26-1.36 | 1.26 | 1.22-1.32 |
|  | SA → N | 1.11 | 1.08-1.14 | 1.09 | 1.06-1.12 |
|  | SA → DP | 1.45 | 1.32-1.60 | 1.28 | 1.15-1.41 |

*Notes:* Model 1: adjusted for sex and birthyear cohort. Model 2: adjusted for sex, birthyear cohort, education, self-rated health, and marital status. E=entry; SA=Sickness absence; N=Not sickness absent; DP=disability pension.
